# Estimating time since infection in early homogeneous HIV-1 samples using a poisson model

**DOI:** 10.1186/1471-2105-11-532

**Published:** 2010-10-25

**Authors:** Elena E Giorgi, Bob Funkhouser, Gayathri Athreya, Alan S Perelson, Bette T Korber, Tanmoy Bhattacharya

**Affiliations:** 1 Los Alamos National Laboratory, Los Alamos, NM 87545, USA; 2 Univeristy of Massachusetts, Amherst, MA 01002, USA; 3 University of Arizona, Tucson, AZ 85721, USA; 4 The Santa Fe Institute, Santa Fe, NM 87501, USA

## Abstract

**Background:**

The occurrence of a genetic bottleneck in HIV sexual or mother-to-infant transmission has been well documented. This results in a majority of new infections being homogeneous, *i.e*., initiated by a single genetic strain. Early after infection, prior to the onset of the host immune response, the viral population grows exponentially. In this simple setting, an approach for estimating evolutionary and demographic parameters based on comparison of diversity measures is a feasible alternative to the existing Bayesian methods (*e.g*., BEAST), which are instead based on the simulation of genealogies.

**Results:**

We have devised a web tool that analyzes genetic diversity in acutely infected HIV-1 patients by comparing it to a model of neutral growth. More specifically, we consider a homogeneous infection (*i.e*., initiated by a unique genetic strain) prior to the onset of host-induced selection, where we can assume a random accumulation of mutations. Previously, we have shown that such a model successfully describes about 80% of sexual HIV-1 transmissions provided the samples are drawn early enough in the infection. Violation of the model is an indicator of either heterogeneous infections or the initiation of selection.

**Conclusions:**

When the underlying assumptions of our model (homogeneous infection prior to selection and fast exponential growth) are met, we are under a very particular scenario for which we can use a forward approach (instead of backwards in time as provided by coalescent methods). This allows for more computationally efficient methods to derive the time since the most recent common ancestor. Furthermore, the tool performs statistical tests on the Hamming distance frequency distribution, and outputs summary statistics (mean of the best fitting Poisson distribution, goodness of fit p-value, etc). The tool runs within minutes and can readily accommodate the tens of thousands of sequences generated through new ultradeep pyrosequencing technologies. The tool is available on the LANL website.

## Background

**Figure 1:**
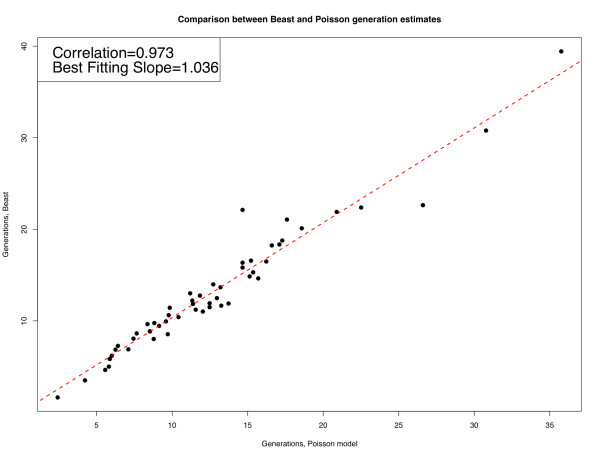
**Correlation between time since MRCA estimated by Beast and the Poisson method**. Estimated time since the MRCA calculated by the Poisson methods for samples from 53 homogeneous HIV-1 infections [3] and how they correlate to the times estimated using the Beast tool [5]. The data is highly correlated: *ρ *= 0.973 and the best fitting slope is 1.036.

The occurrence of a genetic bottleneck in HIV sexual or mother-to-infant transmissions has been well documented [1,2]. This results in about 80% of new infections being homogeneous, *i.e*., initiated by a single genetic strain [3]. Due to the availability of early samples taken at multiple time points from acute HIV subjects, we now know that the viral population grows exponentially during the early phases of infection [4], prior to the onset of the host immune response or significant target cell depletion. Given this simple setting, our goal is to clearly distinguish infections that were initiated by a single strain (homogenous infection) from those where multiple strains entered the host. Furthermore, for those cases where we are able to determine that the infection was indeed homogenous, we seek to estimate the time since the most recent common ancestor (MRCA) given a sample of genetic sequences. In contrast to coalescent and Bayesian inference methods (*e.g*., BEAST [5]), which are based on a simulation of genealogies, we do not simulate the genealogical history of the observed sequences, but rather follow the diversity structure of the entire viral population. While in most settings a forward simulation is not attainable (hence the use of coalescence), our approach becomes not only feasible but extremely simple to realize when modeling a homogeneous infection within a small number of generations from the transmission bottleneck. The increased efficiency of the algorithm (which runs within minutes instead of hours) facilitates the analysis of samples from a large number of subjects [3] and enables the application to massive new data sets that are currently being gathered using pyrosequencing [6]. We have successfully tested our tool in both cases, *i.e*., hundreds of patients and tens of thousands of sequences. These methods are robust against violations of assumptions that do not strongly affect overall diversity. Quantities like the time since the MRCA, when computed by our tool, yield virtually identical results [3,7] to those computed by the coalescent or Bayesian methods, as shown in Figure 1. The tool is available on the LANL website [8]. An extensive explanation of how the tool works and how to format the input data is provided in the *Poisson Fitter Explanation *link available on the tool website [8].

## Implementation

Our basic framework is that of an exponentially growing population following a narrow bottleneck, with lineage-independent mutation rates at all sites and no differential selection among the attested forms. When the resulting diversity is small, almost every change is at a distinct locus, and the pairwise differences between genetic strains, *i.e*., the Hamming distances (HD), follow a Poisson distribution [9]. There is little evidence of recombination in samples from this early stage when care is taken to guard against in vitro recombination [10]. Furthermore, when sites evolve independently starting from a homogeneous infection, homologous recombination does not change the HD distribution nor the overall amount of diversity. It does, however, affect the distribution when these assumptions are violated (unpublished data). The Poisson Fitter tool [8] uses this fact to fit a Poisson distribution to the pairwise HD frequency distribution obtained from the input data. Because a good fit is obtained when the infection is homogeneous and free of differential selection pressure [7], the tool can be used to detect large violations of one or more assumptions of the null model.

### Input data

The Poisson Fitter tool [8] was originally developed to analyze genetic sequences from early HIV-1 infected patients (Fiebig Stage I and II [3]). However, it can be used on any set of highly conserved sequences to test whether the observed diversity could be due to random accumulation of mutations or whether one needs to invoke alternate explanations like early onset of selection or multiple original infecting strains. The tool accepts multiple alignments, obtained through either single genome amplification [10] or deep sequencing [6], but they must be uploaded as one file. All alignments need to be in fasta format and always start with a consensus or reference sequence, which is assumed to be the ancestral strain. More details on how to format the input data is provided on the tool web page [8].

### Controlling for APOBEC enrichment

APOBEC is a host enzyme. During replication it causes mutations that can introduce stop codons and inactivate the virus. These substitutions are recognizable by a *G *→ *A *mutational pattern embedded in the APOBEC3G/F motif [11,12], which is: *GRD*, where *R *is the IUPAC symbol for *G *or *A*, and *D *for *G*, *A *or *T*. Their occurrence is sporadic, often leading to overtly hypermutated sequences [11,12], and thus violating our assumption of a constant mutation rate (see Results and Discussion for an example). Our tool allows for two types of checks: the first method searches for hypermutated sequences, *i.e*., particular sequences that present a statistically significant excess of *G *→ *A *mutations at APOBEC3G/F motifs compared to elsewhere in the sample. The test is performed using the Hypermut tool [13] and the user can select a P-value threshold (default *P *< 0.1). With the second method, an artificial sequence is created, which summarizes all mutations found across the sample, and is then tested for APOBEC enrichment via the Hypermut tool [13]. When the latter is significantly enriched, it indicates enrichment across the sample for mutations embedded in the APOBEC motif, in which case we call the sample overall enriched [14]. The user can choose whether and how to correct for APOBEC enrichment. Two correction methods are provided, which correspond to the two tests discussed above. The first option is to simply remove all sequences that yield a P-value below the threshold. Because this method reduces the number of sequences, we have devised a second option which looks at all *G*'s in the consensus sequence and removes the positions that fall within an APOBEC GRD motif from the entire alignment. This leaves the number of sequences unchanged and provides an unbiased correction. With either option, a new *corrected *alignment will be created, and the tool will proceed to analyze both the uncorrected and the corrected alignments.

### Star phylogeny

When the samples are relatively small, under our assumed model of neutral evolution and rapid exponential growth, all sequences are likely to coalesce to the same founder or MRCA [15]. When this happens, we say that the sample follows a star phylogeny. This has been shown in HIV-1 early homogenous infections [3,16,17]. Under a star-phylogeny scenario, the pairwise HD distribution coincides with the self-convolution of the HD_0 _frequency distribution, where HD_0 _is the Hamming distance from the consensus sequence. When the sequences coalesce at the founder we can use the following mathematical formulation to compute the HD frequencies [7]:

$$Y_{j}=\frac{1}{2}\sum_{i=0}^{j}X_{i}X_{j-i}-\frac{1}{2}\delta_{j,j-i}X_{i}$$

where *δ*_*x, y *_is the Kroenecker delta, and *X_i _*is the number of sequences that are *i *bases from the consensus, and *Y_j _*are the number of pairs of sequences that are different at *j *bases. By comparing these theoretical frequencies *Y_j _*to the observed ones (i.e., the number of pairs that are *j *bases from one another), one can verify whether the star-phylogeny assumption is valid for the tested alignment.

### Fitting the Poisson distribution

A Poisson distribution is fitted to the observed pairwise HD distribution using a maximum likelihood method (see [7] for details). A *χ*^2 ^goodness of fit (GOF) is performed to test whether the HD distribution significantly diverges from a Poisson (small P-values indicate a bad fit). The test takes into account the non-independence of pairwise HD distances by comparing the observed frequencies to the expected ones if the sample were to follow a star phylogeny. Prior to the onset of positive selection, the population is assumed to undergo a rapid expansion during which the basic reproductive number *R*_0 _> 1. Therefore, when the sample yields a GOF P-value above 0.05 (indicating a non-significant divergence from a Poisson distribution), we can estimate the time since the MRCA using the parameters characterizing intrahost HIV evolution. Following Stafford *et al*. [18], we set *R*_0 _= 6 for acute HIV-1 infection samples. We assume a constant mutation rate across lineages, which we fix at an average value of ϵ = 2.16 × 10^-5 ^per site and per replication cycle. This value has been adjusted from what was originally derived for HIV-1 by Mansky *et al*. [19] by considering only base substitutions and ignoring insertions and deletions. However, it has not been corrected for possible extra purifying selection *in vivo*. Using these parameters, we use the mean of the fitted Poisson distribution to infer the time since the MRCA [7].

## Results and Discussion

All the parameters explained in the previous section are computed and included in the output table called "Log Likelihood - Estimated Parameters." This comprises, for each sample: the number of sequences in the sample, the mean and maximum pairwise HD, the mean of the best fitting Poisson distribution, the corresponding time since the MRCA, and the goodness of fit P-value It is important to notice that when the sample meets our model's assumptions, the mean of the best fitting Poisson distribution is in fact the mean pairwise HD of the sample. A second table, called "Convolution Estimates," provides the observed HD frequencies and the estimated ones calculated using equation (1). A more detailed explanation of the parameters is provided in the *Explanation *file on the tool web page.

**Figure 2:**
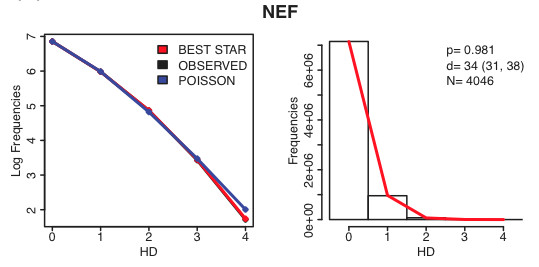
**Example of output graphics for a 454 sample that conformed to the model**. Pairwise HD frequency plots on a logarithmic scale (black, left panel), together with the best fitting Poisson (blue) and the theoretical counts expected if the sample were to follow a star-like phylogeny. The right panel shows the pairwise HD histogram and the best fitting Poisson distribution (red). In the legend we report the GOF P-value (*P *= 0.981), the estimated days since the infections (*d *= 34(31, 38)), and the number of sequences in the sample (*N *= 4046).

Figure 2 shows the graphics obtained by analyzing a fragment of the NEF HIV-1 gene (169 base pairs) from patient CH40 [3,16]. The data have been published [16] and submitted to the NCBI Sequence Read Archive http://www.ncbi.nlm.nih.gov/Traces/sra under accession number SRA020793. This sample was obtained through deep sequencing [6,20] and yielded a little over 4,000 sequences, though our tool can easily handle ten times as many sequences: because it works only with counts of pairwise distances, it can handle samples of almost any reasonable size, though very large jobs will slow the server. The left panel in Figure 2 shows the pairwise HD frequency counts (black), the best fitting Poisson distribution (blue), and the expected counts if it were a star-phylogeny (red) on a logarithmic scale. The fact that the red line and the black line are indistinguishable confirms that the sample follows a star-like phylogeny. Because the Poisson fit is very sensitive to deviations in the upper tail of the distribution, the tool outputs graphics in the logarithmic scale whenever the sample size is above 100; this helps visualize possible deviations at the higher distances. Though the values are discrete, lines are used for better visualization. In the right panel a histogram of the frequency counts is shown together with, in red, the best fitting Poisson distribution. In this case the sample yielded a good fit (*P *= 0.981) and a time to MRCA of 34 days 95% CI = (31, 38).

**Figure 3:**
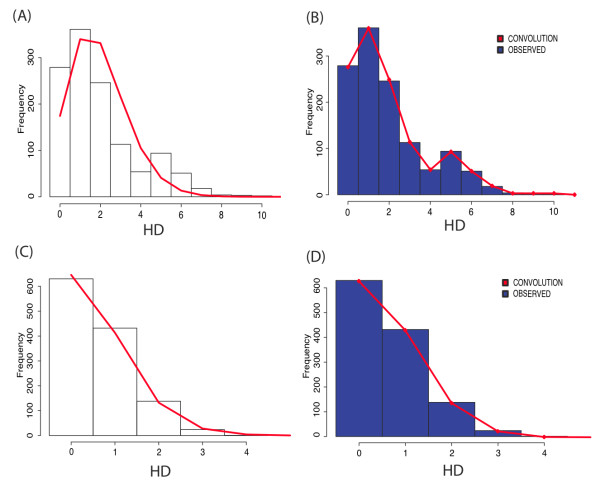
**Example of output graphics for an SGA sample that was enriched for APOBEC mediated substitutions**. HD frequency plots with best fitting Poisson (red line), on the left (panels A and C), and with theoretical star-phylogeny frequencies (red line), on the right (panels B and D). The top panels represent an alignment not corrected for enrichment for APOBEC motifs, whereas the bottom panels represent the same alignment after the G positions that in the consensus are in the APOBEC context have been removed from the alignment. Prior to the correction, the Poisson does not fit the HD frequency distribution (GOF *P *< 0.0001), whereas after the correction the Poisson yields a good fit (GOF *P *= 0.865).

As a second example, Figure 3 shows a sample drawn from single genome amplification sequencing [3,10]. All fifty sequences used for this example are available through Accession Numbers EU575084-133. In this case the original alignment does not yield a good fit to the Poisson distribution (top left panel, red line), but the tool detected APOBEC3G/F mediated hypermutation. By selecting the option to correct for APOBEC signatures by both methods, two more alignments are produced: one where two significantly (*P *< 0.1) hypermutated sequences are removed, and one where instead the alignment position where APOBEC induced mutation could potentially affect results are removed. It is noteworthy that the first type of correction still does not yield a good Poisson fit (GOF *P *< 0.0001). This is because the sample is overall enriched for *G *→ *A*. One can check this by looking at the Hypermut Results Table and noticing that the sequence called *compressedMutations *yields a *P *= 6 × 10^-6 ^for APOBEC enrichment. Therefore, only when all positions with a *G *embedded in a APOBEC3G/F motif are removed, does one finally achieves a good Poisson fit (*P *= 0.865) and a biologically sensible (given the clinical data available for this subject) time estimate of the time since the MRCA of 12 days, 95% CI = (8, 16). This example illustrates how APOBEC enrichment can cause the Poisson fit to fail and hence how it is necessary to isolate the APOBEC induced mutations in order to make sensible estimates on the timing of the infection.

Unlike the example in Figure 2, where a logarithmic scale is used for better visualization, when the sample size is under 100, the star-phylogeny is represented in the manner shown in the panels on the right: the observed pairwise HD frequency counts are shown by the blue histograms, whereas the ones computed theoretically are shown in red. For both APOBEC-corrected and non-corrected samples, the red lines follow the histograms faithfully, which deems both samples as star-like phylogenies.

Both of the examples above obviously meet our model's assumptions of exponential growth with no selection and negligible recombination rate. When one or more assumption is not met the goodness of fit P-value lowers considerably and therefore the time since the MRCA is inaccurate. There are several factors that can cause this to happen: for instance, the infection may be non-homogeneous, the sample may not be "early" enough, or one may have sampled an unlikely early random mutation that distorts the Poisson distribution. When analyzing HIV-1 data, we recommend using samples taken within the first 2-5 weeks of infection, or characterized as Fiebig stage I or II [3]. At later Fiebig stages selection and recombination are clearly observed, and the diversity is controlled by these later selective bottlenecks. The probability of an early stochastic mutation violating the model assumption is calculated in [14], and is typically small.

Finally, we notice that our tool can be applied to subsets of sequences sampled at later time points when there is evidence of a narrow bottleneck. For example, in Fischer et al. [16] we were able to isolate the escape lineages after the immune response had begun and applied the tool to estimate the timing of each lineage. The tool has been used primarily on large HIV-1 data sets [3,16], though it can be used on any population that grows in a similar fashion, as appears to be the case of HCV for instance [21].

## Conclusions

Our tool enables quantitative characterization of acute infection samples and can be usefully applied in large scale vaccine and prophylaxis studies where estimates of the time since the MRCA and/or the timing of the onset of host selection can be extremely informative. The tool can rapidly detect whether the mutational distribution in a set of HIV sequences is consistent with a star phylogeny and/or a Poisson model, indicative of a population evolving from a single ancestor, with lineage independent mutations under no differential selection of surviving forms. If the model is violated, the tool automatically evaluates whether this is a consequence of APOBEC mediated substitutions. When the model is satisfied, it can be be used to estimate times to the most recent common ancestor of the lineage, rapidly providing timing estimates that are in good accord with coalescent methods. The speed and simplicity of the algorithm enables it to be applied to massive data sets obtained through ultra-deep sequencing methods [6,20]. We have used it on tens of thousands of sequences [16] to estimate the time since the MRCA and also, at later time points, to estimate the timing of escape lineages, which arose after the onset of selection. The tool can be used on any other population which presents similar evolutionary patterns [21].

## Authors' contributions

BTK was the project PI, provided the APOBEC modeling concept, and helped draft the manuscript; BF built the web interface; GA provided codes to be included in the tool; both BF and GA helped with the web tool development and programming; ASP, BTK, and TB provided the theoretical frame for the study and helped drafting the manuscript; EEG provided codes for the tool, contributed to the analysis, and drafted the manuscript. All authors read and approved the final manuscript.

## References

[B1] Wolinsky SM Wike CM Korber BT Hutto C Parks WP Selective transmission of human immunodeficiency virus type-1 variants from mothers to infants Science 1992 255 1134 1137 10.1126/science.1546316 1546316

[B2] Delwart EL Magierowska M Royz M Foley B Peddada L Homogeneous quasispecies in 16 out of 17 individuals during very early HIV-1 primary infection AIDS 2001 15 1 7 10.1097/00002030-200101050-00003 11807302

[B3] Keele BF Giorgi EE Salazar-Gonzalez JF Decker JM Pham KT Identification and characterization of transmitted and early founder virus envelopes in primary HIV-1 infection Proc Natl Acad Sci USA 2008 105 7552 7557 10.1073/pnas.0802203105 18490657 PMC2387184

[B4] Ribeiro RM Qin L Chavez LL Li D Self SG Perelson AS Estimation of the initial viral growth rate and basic reproductive number during acute HIV-1 infection J of Virology 2010 12 6096 102 10.1128/JVI.00127-10 PMC2876646 20357090

[B5] Drummond AJ Rambaut A BEAST: Bayesian evolutionary analysis by sampling trees BMC Evol Biol 2007 7 214 17996036 10.1186/1471-2148-7-214 PMC2247476

[B6] Margulies M Egholm M Altman WE Attiya S Bader JS Genome sequencing in microfabricated high-density picolitre reactors Nature 2005 437 376 380 16056220 10.1038/nature03959 PMC1464427

[B7] Lee HY Giorgi EE Modeling sequence evolution in acute HIV-1 infection J Theor Biol 2009 2 261 341 360 10.1016/j.jtbi.2009.07.038 PMC2760689 19660475

[B8] Poisson fitter http://www.hiv.lanl.gov/content/sequence/POISSON_FITTER/poisson_fitter.html

[B9] Slatkin M Hudson RR Pairwise comparisons of mitochondrial DNA sequences in stable and exponentially growing populations Genetics 1991 129 555 562 1743491 10.1093/genetics/129.2.555 PMC1204643

[B10] Hahn S Zhong XY Troeger C Burgemeister R Gloning K Holzgreve W Current applications of single-cell PCR Cell Mol Life Sci 2000 57 96 105 10.1007/s000180050501 10949583 PMC11147117

[B11] Bourara K Liegler TJ Target cell APOBEC3G can induce limited G-to-A mutation in HIV-1 PLoS Path 2007 3 1477 1485 10.1371/journal.ppat.0030153 PMC2042017 17967058

[B12] Simon V Zennou V Natural variation in Vif: differential impact on APOBEC3G/3F and a potential role in HIV-1 diversification PLoS Path 2005 1 1 e6 10.1371/journal.ppat.0010006 PMC1238741 16201018

[B13] HYPERMUT http://hiv-dev.lanl.gov:8081/content/sequence/HYPERMUT/hypermut.html

[B14] Wood N Bhattacharya T Keele BF Giorgi EE Liu M Gaschen B Daniels M Ferrari G Haynes BF McMichael A Shaw GM Hahn BH Korber B Seoighe C HIV evolution in early infection: selection pressures, patterns of insertion and deletion, and the impact of APOBEC PLoS Path 2009 5 5 414 10.1371/journal.ppat.1000414 PMC2671846 19424423

[B15] Wakeley J Coalescent Theory, an Introduction 2008 Roberts and Co

[B16] Fischer W Bhattacharya T Keele BF Giorgi EE Hraber PT Perelson AS Shaw GM Korber BT Rapid mutational escape from cytotoxic T-cell responses in acute HIV-1 infection--an ultra-deep view PLoS ONE in press

[B17] Salazar-Gonzalez JF Salazar MG Keele BF Learn GH Giorgi EE Genetic identity, biological phenotype, and evolutionary pathways of transmitted/founder viruses in acute and early HIV-1 infection Journal of Experimental Medicine 2009 206 6 1273 1289 10.1084/jem.20090378 19487424 PMC2715054

[B18] Stafford MA Corey L Cao Y Daar ES Ho DD Modeling plasma virus concentration during primary HIV infection J Theor Biol 2000 203 285 301 10.1006/jtbi.2000.1076 10716909

[B19] Mansky LM Temin HM Lower in vivo mutation rate of human immunodeficiency virus type 1 than that predicted from the fidelity of purified reverse transcriptase J Virol 1995 69 5087 5094 7541846 10.1128/jvi.69.8.5087-5094.1995 PMC189326

[B20] Tsibris AM Korber B Arnaout R Russ C Lo CC Leitner T Gaschen B Theiler J Paredes R Su Z Hughes MD Gulick RM Greaves W Coakley E Flexner C Nusbaum C Kuritzkes DR Quantitative deep sequencing reveals dynamic HIV-1 escape and large population shifts during CCR5 antagonist therapy in vivo PLoS ONE 2009 5 4 5683 10.1371/journal.pone.0005683 PMC2682648 19479085

[B21] Wang GP Sherrill-Mix SA Chang KM Quince C Bushman FD Hepatitis C virus transmission bottlenecks analyzed by deep sequencing J Virol 2010 84 12 6218 28 10.1128/JVI.02271-09 20375170 PMC2876626

